# Suicidality among older Australian adults

**DOI:** 10.3389/fpubh.2022.992884

**Published:** 2023-01-09

**Authors:** Britt Klein, Kerrie Shandley, Suzanne McLaren, Lisa Clinnick, Huy Van Nguyen

**Affiliations:** ^1^Health Innovation and Transformation Centre, Federation University Australia, Ballarat, VIC, Australia; ^2^Biopsychosocial and eHealth Research and Innovation (BeRI) Hub, Federation University Australia, Ballarat, VIC, Australia; ^3^Faculty of Business, Justice and Behavioural Sciences, School of Psychology, Charles Sturt University, Bathurst, NSW, Australia

**Keywords:** suicidality, older adults, social connection, community engagement, risk factors, mental health, physical health

## Abstract

**Background:**

Vulnerability to suicidality is a concern among older adults, particularly as this proportion of the population is growing. Determining what factors contribute to suicidality will help to create a framework for understanding and assessing suicidal risk among older adults and developing effective treatments. This study examined suicidality among older Australian adults.

**Methods:**

This study forms part of a larger study to trial a survey to collect cross-sectional data on the mental and physical health of older Australian adults across time. One hundred and fourteen Australian residents aged 65 years and over completed an anonymous survey online or by returning a paper-and-pencil version of the survey by post. The survey took approximately 25 min to complete and comprised of (1) sociodemographic questions (e.g., age, gender, education), (2) validated questionnaires measuring depression, general anxiety, psychological distress, insomnia, substance dependence, problem gambling, and stress, and (3) mental and physical health and wellbeing items (e.g., religiosity, assistance with daily tasks, and mental health service usage in the last 12-months). The dependent variable, suicidality, was measured by asking participants whether they had ever seriously thought about committing suicide.

**Results:**

Associations with suicidality were analyzed using Chi-squares and independent samples *t*-tests. The results found suicidality to be significantly associated with lower levels of satisfaction with the frequency of seeing and/or communicating with friends, and inadequate levels of community engagement.

**Conclusion:**

The results of this survey reinforce the importance of social connectedness as a central and significant protective factor against suicidality among older adults.

## Introduction

The Australian population is aging rapidly. In 20 years (2000–2020) the proportion of people aged 65 years and over increased by 3.9%, from 12.4 to 16.3% (1). In 2020, suicide was the 10th leading cause of death for men and 22nd leading cause of death for women (2). The highest age-specific suicide rate was recorded for men aged 85 years and older (36.2 deaths per 100,000 persons). Comparatively for women, the age-specific suicide rate for those aged 85 years and over was 6.2 per 100,000 persons.

A systematic literature review (3) identified that suicidal behavior among older adults aged 65 years and over was associated with functional disability and a variety of conditions (e.g., malignant diseases, neurological disorders, pain, liver disease, arthritis). The review included 59 quantitative studies across four continents. A further six qualitative studies from three continents unpacked the results of the quantitative studies finding common themes of illness, disability, pain, feelings of being a burden, lack of dignity, independence and sense of usefulness, and lack of pleasure with living to be common themes precipitating suicidal thoughts, attempts and completed suicides. Furthermore, mental health conditions (i.e., dementia, depression, anxiety) have been positively correlated with the prevalence of suicide among older adults (4). In addition, there is also a tendency for health professionals not to routinely assess for mental health conditions amongst older adults and this can result in the under-identification and under-reporting of actual rates (5), potentially increasing the risk of suicide.

Suicidality among older adults has also been associated with a range of other mental health issues, such as insomnia (6), substance abuse (7), and gambling (8), Determining what factors contribute to suicidality will help to create a framework for understanding and assessing suicidal risk among older adults, and ultimately developing and administering effective treatments.

The aim of this study was to conduct a survey of older Australian adults (aged 65 years and over) to examine suicidality across sociodemographic variables, depression, anxiety, psychological distress, insomnia, substance dependence, problem gambling, stress, and mental health and physical well-being items.

## Methods

### Procedure and participants

This paper reports on data collected as part of a larger study to trial a survey to collect cross-sectional data on the mental and physical health of older Australian adults across multiple timepoints. Recruitment for this study was conducted nationally, with all Australian residents aged 65 years and older eligible to take part by completing an anonymous survey online or returning a paper-and-pencil version of the survey by post. Following ethics approval, the study was advertised *via* social and printed media, flyers on community notice boards, and in aged care residential facilities. The survey took approximately 25 min to complete. A total of 168 older adults consented to take part in the study, six participants were ineligible as they resided outside of Australia and were removed from the sample for analysis. A further 48 participants failed to complete an essential question for analysis (whether they had ever seriously thought about committing suicide) and were subsequently also removed from analysis. This left a total sample size of 114. Participant characteristics of this sample are presented in Table 1.

**Table 1:** Participant characteristics.

**Variables (*N* = 114)**	**n (%)**	**Mean (SD)**
Age		71.59 (5.33)
**Gender identity**		
Male	43 (37.7)	
Female	71 (62.3)	
**Highest level of education completed**		
Year 12 or lower	32 (28.1)	
TAFE	32 (28.1)	
University	50 (43.9)	
**Gross annual income**		
$0–19,999	22 (19.3)	
$20,000–39,999	51 (44.7)	
$40,000–79,000	33 (28.9)	
$80,000+	8 (7.0)	
**Country of birth**		
Australia	72 (63.2)	
Overseas	41 (36.0)	
Not answered	1 (0.9)	
**Relationship status**		
Separated/divorced/widowed/single	46 (40.4)	
Married/partnered	68 (59.6)	
**Living setting**		
Aged care facility/Retirement village	14 (12.3)	
Community (independently, with partner or other family member/s)	100 (87.7)	
**Sexually Active (last 12 months)**		
No	56 (49.1)	
Yes	41 (36.0)	
Prefer not to say/Not answered	17 (14.9)	
**Have you** ***ever*** **seriously thought about committing suicide?**		
No	92 (80.7)	
Yes	22 (19.3)	
**Have you seriously thought about committing suicide at any time in the past 12 months?** ^*^		
No	16 (72.7)	
Yes	6 (27.3)	
**Have you** ***ever*** made a plan for committing suicide?^*^		
No	8 (36.4)	
Yes	7 (31.8)	
No response	7 (31.8)	
**Have you made a plan for committing suicide in the past 12 months?** ^*^		
No	4 (18.2)	
Yes	3 (13.6)	
No response	15 (68.2)	
**Have you** ***ever*** attempted suicide?^*^		
No	11 (50.0%)	
Yes	3 (13.6%)	
No response	8 (36.4%)	

* Only participants (N = 22) who responded “Yes” to the question “Have you ever seriously thought about committing suicide” were asked these questions.

### Measures

The survey included sociodemographic items, validated measures, and items relating to the mental and physical health and well-being of the participants.

Sociodemographic information collected included age, gender, highest level of education completed, gross income per annum, country of residence and birth, living setting, relationship status, and whether the participant had been sexually active in the last 12 months.

Patient Health Questionnaire - 9 [PHQ-9; (9)]. The PHQ-9 is a nine-item depression subscale of the Patient Health Questionnaire (10). The questionnaire is based on the diagnostic criteria from the Diagnostic and Statistical Manual, fourth edition (11). Questions are based on symptoms experienced in the previous 2 weeks, with questions answered on a four-point scale from 0 = “Not at all” to 3 = “Nearly every day” and then summed to obtain a score from 0–27.

Generalized Anxiety Disorder - 7 [GAD-7; (12)]. The GAD-7 is a seven-item screening tool for GAD. Questions are based on symptoms experienced in the previous two weeks with items answered on a four-point scale from 0 = “Not at all” to 3 = “Nearly every day”. Items are summed to obtain a score from 0–21.

Kessler 6 [K6; (13)]. The K6, an abridged version of the K10, is a six-item screening tool to provide a general measure of psychological distress. Questions ask about depressive- and anxiety-related symptomology during the previous 30 days. For the Australian version of the K6, symptoms are rated on a five-point scale from 1 = “None of the time” to 5 = “All of time” with scores summed to obtain a score from 6–30.

Insomnia Severity Index [ISI; (14)]. The ISI is a seven-item measure assessing the nature, severity, and impact of both nighttime and daytime components of insomnia. Questions are based on sleep-related behaviors across the prior 2 weeks. Each question is answered on a five-point Likert scale from 0 = “None” to 4 = “Very severe” for the first three questions, (1) difficulty falling asleep, (2) difficulty staying awake, and (3) problems waking up too early; 0 = “Very satisfied” to 4 = “Very dissatisfied” for (4) satisfaction with current sleep pattern; 0 = “Not noticeable” to 4 = “Very much noticeable” for (5) noticeability of sleep problem to others; 0 = “Not worried” to 4 = “Very much worried” for (6) degree of worry/distress about current sleep problem; and 0 = “Not interfering” to 4 = “Very much interfering” for (7) extent that sleep problem interferes with daily functioning. Responses to the seven-items are summed to obtain a total score from 0–28, with scores interpreted as: 0–7 = No clinically significant insomnia, 8–14 = Subthreshold insomnia, 15–21 = Clinical insomnia (moderate severity), and 22–28 = Clinical insomnia (severe).

CAGE Adapted to Include Drugs [CAGE-AID; (15)]. The CAGE-AID is a four-item tool adapted from the CAGE to screen for alcohol and other drug problems. Each question is given a “yes” or “no” response, with one or more “yes” responses considered an indication of possible substance use or abuse.

Problem Gambling Severity Index [PGSI; (16)]. The PGSI is a nine-item measure of common signs for risk of problem gambling. Questions are based on the prior 12 months and answered on a four-point scale from 0 = “Never” to 3 = “Almost always”, with scores summed to obtain a total score from 0–27. The total score is interpreted as: 0 = Non-problem gambler, 1–2 = Low-risk gambler, 3–7 = Moderate-risk gambler, and 8 or above = Problem gambler.

The Social Readjustment Rating Scale [SRRS; (17)]. The SRRS measures the amount of stress experienced over the prior 12 months and the risk of future illness. Participants are presented with a list of major stressful life events and asked to state whether they experienced that event within the last 12 months. Each event has been designated a score of up to 100 points, for example, death of a spouse or child = 100 points, marriage = 50 points, and children leaving home = 29 points. The scores for endorsed items are summed to obtain a total score where 0–150 = a low susceptibility to stress-induced health breakdown, 150–300 = a 50% (moderate) chance of health breakdown in the next 2 years, and 300 or more = an 80% (high) chance of health breakdown in the next 2 years.

Participants were asked to answer a series of questions related to their mental health and physical well-being. Questions included: (a) whether they had ever seriously thought about dying by suicide (yes/no); (b) whether they had ever been a smoker (yes/no); (c) how religious they are (1 = not at all religious to 11 = extremely religious); how satisfied they were with the frequency of seeing and communicating with (d) family and (e) friends (1 = No nearly enough to 11 = Very satisfied with how often); (f) their satisfaction with level of community engagement (1 = Not at all, to 5 = Very much); overall satisfaction with (g) physical health, (h) mental health, and (i) quality of life (1 = Excellent to 5 = poor); whether they had experienced symptomatology relating to (j) social phobia, (k) specific phobia, (l) post-traumatic stress disorder (PTSD), (m) obsessive-compulsive disorder (OCD), (n) panic disorder (1 = yes, 2 = sometimes, 3 = not now, but used too, 4 = no, never have); (o) whether they needed assistance with daily tasks because of physical illness or disability (1 = yes, 2 = sometimes, 3 = no); whether they (p) had ever experienced a direct blow to the head, and (q) were physically hit/smacked as a child (1 = no, never, 2 = yes, but only brief exposure, 3 = yes, prolonged exposure); (r) whether they had experienced any physical conditions from a provided list (i.e., hypertension, diabetes, osteoporosis); and (s) whether they had accessed any services from a provided list (i.e., psychologist, pharmacist, telephone service) in the past 12 months for a mental health condition.

### Results

All analyses were performed using IBM SPSS (v.28). The dependent variable was suicidality, specifically, whether the participant had seriously considered suicide at any point in their lifetime. Suicidality was examined against socio-demographics, validated measures, and mental and physical health and well-being items using Chi-squares and independent samples *t*-tests, corresponding to categorical and continuous/interval variables respectively. The *p*-value was set conservatively at 0.01 due to the number of analyses conducted. The results are presented in Table 2.

**Table 2:** Suicidality by sociodemographic, validated measures, and mental and physical health and well-being items.

**Variables (*N* = 114)**	**Suicidality**		***p*-value**
	**No**	**Yes**	
**Sociodemographics**			
Age, mean (*SD*)^a^	71.90 (5.52)	70.27 (4.30)	0.20
**Gender identity**			
Male	33 (76.7)	10 (23.3)	
Female	59 (83.1)	12 (16.9)	0.40
**Highest level of education completed**			
Year 12 or lower	24 (75.0)	8 (25.0)	
TAFE	26 (81.3)	6 (18.8)	
University	42 (84.0)	8 (16.0)	0.60
**Gross annual income**			
$0–19,999	20 (90.9)	2 (9.1)	
$20,000–39,999	38 (74.5)	13 (25.5)	
$40,000–79,000	27 (81.8)	6 (18.2)	
$80,000+	7 (87.5)	1 (12.5)	0.39
**Country of birth**			
Australia	61 (84.7)	11 (15.3)	
Overseas	31 (75.6)	10 (24.4)	0.23
**Relationship status**			
Separated/divorced/widowed/single	36 (78.3)	10 (21.7)	
Married/partnered	56 (82.4)	12 (17.6)	0.59
**Living setting**			
Aged care/Retirement village	12 (85.7)	2 (14.3)	
Community (independently or with family)	80 (80.0)	20 (20.0)	0.61
**Sexually active (last 12 months)**			
No	41 (73.2)	15 (26.8)	
Yes	36 (87.8)	5 (12.2)	0.08
**Validated questionnaires**			
PHQ-9 (depression), mean (*SD*)^a^	3.39 (4.29)	4.18 (4.24)	0.44
GAD-7 (general anxiety), mean (*SD*)^a^	2.43 (3.54)	2.55 (3.14)	0.89
K6 (psychological distress), mean (*SD*)^a^	8.40 (4.56)	10.23 (5.84)	0.11
ISI (insomnia), mean (*SD*)^a^	7.04 (4.83)	8.00 (3.99)	0.44
**CAGE-AID (substance dependence)**			
No	46 (88.5)	6 (11.5)	
Yes	24 (70.6)	10 (29.4)	0.04
**PGSI (problem gambling)**			
Low risk gambler	2 (66.6)	1 (33.4)	
Moderate risk gambler	2 (100.0)	0 (0.0)	0.39
**SRRS (stress)**			
Low susceptibility	91 (81.8)	18 (18.2)	
Moderate susceptibility	10 (71.4)	4 (28.6)	0.36
**Mental health and physical well-being**			
**Smoker (current or past)**			
No	58 (86.6)	9 (13.4)	
Yes	33 (71.7)	13 (28.3)	0.05
Religiosity, mean (*SD*)^a^	4.79 (3.35)	4.09 (3.74)	0.39
Satisfaction with seeing/communicating with family, mean (*SD*)	6.87 (3.12)	5.76 (3.71)	0.16
Satisfaction with seeing/communicating with friends, mean (*SD*)	7.78 (2.83)	5.24 (3.36)	0.00
**Satisfaction with community engagement**			
Inadequate (not at all/a little)	9 (52.9)	8 (47.1)	
Adequate (somewhat/much/very much)	83 (85.6)	14 (14.4)	0.00
**Overall satisfaction with physical health**			
Unsatisfactory (poor/fair)	20 (76.9)	6 (23.1)	
Satisfactory (good/very good/excellent)	71 (81.6)	16 (18.4)	0.60
**Overall satisfaction with mental health**			
Unsatisfactory (poor/fair)	9 (60.0)	6 (40.0)	
Satisfactory (good/very good/excellent)	83 (83.8)	16 (16.2)	0.03
**Overall satisfaction with quality of life**			
Unsatisfactory (poor/fair)	10 (76.9)	3 (23.1)	
Satisfactory (good/very good/excellent)	82 (81.2)	19 (18.8)	0.71
**Social phobia**			
No	70 (84.3)	13 (15.7)	
Yes	22 (71.0)	9 (29.0)	0.11
**Specific phobia**			
No	51 (75.0)	17 (25.0)	
Yes	41 (89.1)	5 (10.9)	0.06
**PTSD**			
No	65 (82.3)	14 (17.7)	
Yes	26 (78.8)	7 (21.2)	0.67
**OCD**			
No	74 (82.2)	16 (17.8)	
Yes	18 (75.0)	6 (25.0)	0.43
**Panic disorder**			
No	82 (80.4)	20 (19.6)	
Yes	10 (83.3)	2 (16.7)	0.81
**Assistance with daily tasks**			
No	75 (82.4)	16 (17.6)	
Yes (yes/sometimes)	12 (66.7)	6 (33.3)	0.13
**Direct blow to the head**			
No	61 (82.4)	13 (17.6)	
Yes (brief or prolonged exposure)	25 (75.8)	8 (24.2)	0.42
**Physically hit/smacked as a child**			
No	30 (85.7)	5 (14.3)	
Yes (brief or prolonged exposure)	56 (77.8)	16 (22.2)	0.33
**Physical illness**			
None	25 (89.3)	3 (10.7)	
1–2 conditions	36 (78.3)	10 (21.7)	
≥3 conditions	31 (77.5)	9 (22.5)	0.41
**Access to mental health services (last 12 months)**			
No	45 (86.5)	7 (13.5)	
Yes	40 (74.1)	14 (25.9)	0.11

a t-test-based p-values, the remaining p-values are based on Chi-square statistics.

The results indicate that two variables were significant at the 0.01 level with suicidality associated with lower levels of satisfaction with the frequency of seeing and/or communicating with friends, and suicidality more than three times higher among participants who reported inadequate levels of community engagement compared to those who reported adequate levels of engagement.

## Discussion

This study explored what variables were associated with suicidality among older Australian adults. Suicidality was examined across a series of sociodemographic variables, validated questionnaires, and mental and physical health and well-being items. Suicidality was found to be significantly associated with a dissatisfaction with the frequency of seeing and/or communicating with friends, and inadequate levels of community engagement.

The two variables highlight the importance of social connection where suicidality is concerned, the impact of which is emphasized within the Interpersonal Theory of Suicide (18). This theory posits that suicide will only occur if an individual has both the desire to die by suicide and the ability to do so. The desire to die is comprised of two psychological states, a low sense of belonginess or social isolation, and a perceived burdensomeness. In contrast, strong social support may have a protective effect against stressors in later life that increase one's vulnerability to suicidal risk factors [e.g., (19, 20)].

It could be argued that dissatisfaction with the frequency of seeing and/or communicating with family should similarly be significant. However, the nature of an obligatory relationship with family members and voluntary relationship with friends may have a differential effect on one's mental health and well-being. For example, Huxhold and Miche (21) found social activities with friends may become more important as one ages and creates a buffer against the negative effects of aging. Furthermore, Gallant and Spitze (22) found that in comparison to family members, social networks had more positive than negative influences over the management of chronic illness among older adults, such as disease management, decision-making about chronic illness, and psychosocial coping. Consequently, family members may play a more moderate role in suicidal risk among older adults than that of friends.

It was surprising that there were no further significant associations with suicidality. However, the results for the questionnaires measuring depression, general anxiety, psychological distress, insomnia, and stress suggest this was a relatively healthy cohort of older Australian adults. Although suicidality among our sample was above the worldwide range, 19.3 vs. 2.6–17%; (23). The results of the study may therefore not provide a good reflection of the general populace of older adults. Indeed, in comparison to Australian population data, our sample is comprised of a greater proportion of females [62.3 vs. 53%; (24)], a higher proportion of individuals living in cared accommodation (12.3 vs. 5.2%) and is more highly educated [43.9 vs. 12% had completed a bachelor's degree or higher, (24, 25)]. Consequently, the data should be viewed with caution. The results of the study are also limited by the small sample size and the likely impact this had on the statistical power of the analyses to detect significant results. The sample size also limited the capacity to conduct more sophisticated analyses to examine the relationship between the dependent and independent variables. Furthermore, given the target sample, we may have been better served administering the Geriatric Depression Scale (26) over the PHQ9 to measure depression. Nonetheless, the results underscore the importance of social connection as a potential protective factor against suicidality among older Australian adults.

Going forward, as part of the larger project intended to create a database of cross-sectional data on the mental and physical health and well-being of older Australian adults, we will seek to collaborate with experts in population health, geriatrics, and biostatistics. We will aim to develop a framework protocol to incorporate how best to refine the survey, identify potential short- and longer-term research questions, analyze the data, recruit a larger more representative sample, and report the findings.

## Data availability statement

The datasets presented in this article are not readily available because the unidentified raw data supporting the conclusions of this study will be made available upon reasonable request to the corresponding author and following Federation University HREC ethical approval to do so. Requests to access the datasets should be directed to BK, b.klein@federation.edu.au.

## Ethics statement

The studies involving human participants were reviewed and approved by Federation University Human Research Ethics Committee. Informed consent for participation was obtained for this study in accordance with the National Legislation and the Institutional Requirements.

## Author contributions

All authors listed have made a substantial, direct, and intellectual contribution to the work and approved it for publication.
